# Risk of Malignant Neoplasm in Patients with Incident Rheumatoid Arthritis 1980–2007 in relation to a Comparator Cohort: A Population-Based Study

**DOI:** 10.1155/2016/4609486

**Published:** 2016-08-17

**Authors:** Shafay Raheel, Cynthia S. Crowson, Kerry Wright, Eric L. Matteson

**Affiliations:** ^1^Division of Rheumatology, Mayo Clinic College of Medicine, Rochester, MN 55905, USA; ^2^Department of Health Sciences Research, Mayo Clinic College of Medicine, Rochester, MN 55905, USA

## Abstract

*Objective.* To determine whether the incidence of malignancy is increased in patients with rheumatoid arthritis (RA) compared to a matched comparison cohort and to identify risk for any individual malignancy in RA.* Methods.* A cohort of 813 Olmsted County, Minnesota, residents who first fulfilled 1987 ACR criteria for RA in 1980–2007 was previously identified by medical record review. Medical records of 813 RA cases and a comparison cohort of age and sex matched Olmsted County residents without RA were evaluated retrospectively for cancer occurrence. Patients in both cohorts were followed until death, migration from Olmsted County, or 12/31/2014.* Results.* The RA and non-RA cohorts (mean age at incidence/index date: 55.9 [SD: 15.7] years; 68.4% females in both cohorts) were followed on average of 14.1 (SD: 7.7) and 14.9 (SD: 8.1) years, respectively. Prior to RA incidence/index date, 52 RA patients and 66 non-RA subjects had malignancies excluding NMSC (*p* = 0.21). During follow-up, significantly more malignancies occurred in patients with RA (*n* = 143) than in comparator subjects (*n* = 118; hazard ratio: 1.32; *p* = 0.027). Inclusion of NMSC obviated this difference.* Conclusion.* After excluding NMSC, there was a small to moderately increased risk of malignancies in patients with RA. Cancer surveillance is imperative in all patients with RA.

## 1. Introduction

Patients with systemic rheumatic diseases, particularly rheumatoid arthritis (RA), systemic lupus erythematosus, systemic sclerosis, and idiopathic inflammatory myopathies, are at increased risk of developing malignancies. This risk is related to the pathobiology of the underlying rheumatic diseases including the inflammatory burden, immunological defects, and personal and environmental exposure such as smoking and some viral infections [1]. However, the occurrence of cancer among patients with RA in a nonreferral community-based population has not been thoroughly examined, especially with respect to nonmelanoma skin cancer (NMSC).

A number of studies have shown RA and RA diseases activity as pathogenic factors in the development of lymphoma [2, 3]. Some studies have shown an increased risk of lung cancer and decreased risk of colorectal cancer in patients with RA [4, 5]. The purpose of this study is to evaluate the occurrence of cancer both prior to and after diagnosis of RA in a population-based cohort of patients with RA and compare the occurrence to an age- and sex-matched comparison cohort without RA from the same geographical area, as well as to assess the risk factors for development of cancer among patients with RA.

## 2. Materials and Methods

### 2.1. Patient Cohort

This retrospective, population-based study was conducted using the resources of the Rochester Epidemiology Project, a medical records linkage system that allows ready access to the complete (inpatient and outpatient) medical records from all community medical providers [6]. An inception cohort of all cases of RA diagnosed between January 1, 1980, and December 31, 2007, among Olmsted County, Minnesota, residents ≥18 years of age was previously assembled using the resources of the Rochester Epidemiology project [7]. Incidence date was defined as the earliest date at which the patient fulfilled at least 4 of the 7 American College of Rheumatology 1987 classification criteria for RA [8]. A comparison cohort of Olmsted County residents without RA with similar age, sex, and calendar year was also previously identified [9]. The index date for each non-RA subject was defined as the RA incidence date of the corresponding patient with RA. The institutional review boards of the Mayo Clinic and the Olmsted Medical Center approved this study.

For both the RA and comparator cohorts, cancer diagnoses were retrieved from the Mayo Clinic Cancer Registry (all malignancies except NMSC) and NMSC were abstracted from the medical charts using a standardized abstraction form. Cancer diagnoses from both before and after RA diagnosis were collected. Cancer categories included head/neck, gastric, pancreatic, liver, colon/rectal, other digestive, lung, other thorax, bone, soft tissue, skin (subdivided into melanoma and NMSC), breast, ductal carcinoma in situ, ovarian, other gynecologic, prostate, kidney, bladder, other genitourinary, ophthalmic, central nervous system, lymphoma, leukemia, multiple myeloma, myeloproliferative syndrome, myelodysplastic syndrome, and other.

The information on RA characteristics included RF status, erythrocyte sedimentation rate (ESR) at RA incidence, large joint swelling, joint erosions/destructive changes on radiographs, joint surgeries (i.e., arthroplasty and synovectomy), and extra-articular manifestations of RA (ExRA). ExRA were classified according to the criteria used in our previous studies [10]. Severe ExRA included pericarditis, pleuritis, Felty's syndrome, glomerulonephritis, vasculitis, peripheral neuropathy, scleritis, and episcleritis [11]. Data regarding start and stop dates for use of systemic glucocorticoids (e.g., oral/parental/intraarticular forms of prednisone, prednisolone, methylprednisolone, hydrocortisone, and dexamethasone), disease-modifying antirheumatic drugs (DMARDs) (methotrexate, other DMARDs), and biologic response modifiers (antitumor necrosis factor alpha [anti-TNF*α*] agents, anakinra, abatacept, and rituximab) were collected in all patients. Smoking status was categorized as never, current, or former.

### 2.2. Statistical Analysis

Descriptive statistics were used to summarize data of the RA and comparator groups. The cumulative incidence of malignancy adjusted for the competing risk of death was estimated for both cohorts. This method, although similar to the Kaplan-Meier method, better accounts for patients who die before experiencing malignancy. Patients and comparator subjects with previous malignancy were excluded from analysis. These analyses were completed both as an overall estimate and by cancer type. Cox proportional hazard models were used to examine the differences between cohorts, as well as the association between patient characteristics (age, time since RA diagnosis, etc.) and the rate of development of malignancy within the RA cohort, and to assess trends in malignancy over time. Time-dependent covariates were used to model risk factors that develop over time. Analyses were performed using SAS version 9.4 (SAS Institute, Cary, NC, USA) and R 3.1.1 (R Foundation for Statistical Computing, Vienna, Austria).

## 3. Results

There were 813 incident cases of RA identified. The mean age of diagnosis was 55.9 (SD: 15.7) years with 42 (68.4%) females. The mean length of follow-up after diagnosis was 14.1 (SD: 7.7) years. The majority of the patients in this study were Caucasian, 93.6% in the RA group and 94.8 in the non-RA group. In the non-RA comparator cohort the number of patients and mean age at index date were the same as for the RA cohort. The mean length of follow-up in the non-RA cohort was 14.9 (SD: 8.1) years. There were 21.9% and 17.7% current smokers in the RA and non-RA cohort, respectively. Prior to RA incidence/index date, 52 patients with RA and 66 non-RA subjects had malignancies excluding NMSC (*p* = 0.21). Including NMSC, there were 79 patients in the RA cohort and 108 in the non-RA cohort with malignancies (*p* = 0.024), as there were fewer NMSC in the RA cohort than in the non-RA cohort prior to RA incidence/index date (*n* = 31 versus 51, *p* = 0.018).

### 3.1. Risk of Malignancy in RA Compared to Non-RA

**Figure 1:**
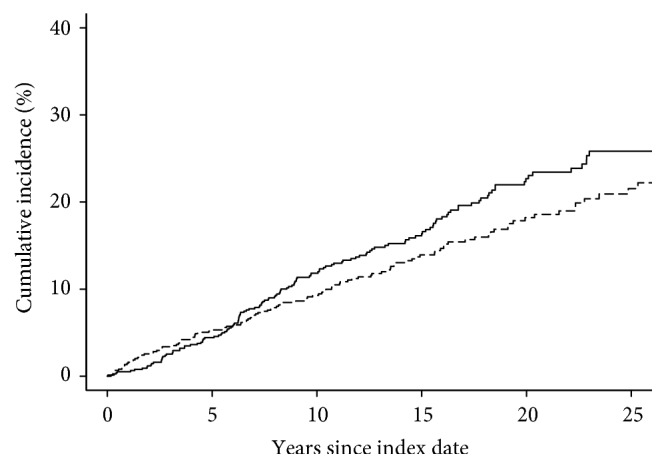
Cumulative incidence of any malignancy excluding nonmelanotic skin cancers among Olmsted County, Minnesota, residents with rheumatoid arthritis (solid line) compared to those without rheumatoid arthritis (dashed line).

A total of 143 patients with malignancies excluding NMSC were detected in the RA cohort, compared with 118 patients experiencing malignancies in the general population comparator cohort. The hazard ratio (HR) of any malignancy excluding NMSC was 1.32 (95% confidence interval [CI]: 1.03, 1.68; Table 1). The cumulative incidence of any malignancy excluding NMSC at 10 years after RA incidence/index date was 11.8% (standard error [SE] 1.2%) among the RA compared to 9.3% (SE: 1.1%) among the non-RA. There was no apparent difference in the cumulative incidence of any malignancy excluding NMSC during the first 5 years after RA incidence/index date (Figure 1). Including cases of NMSC, the total number of patients with malignancies in the RA cohort was 194, compared with 179 in the general population group. The HR of any malignancy including NMSC for RA compared to non-RA did not reach statistical significance (HR: 1.13; 95% CI: 0.92, 1.38).

The incidence of hematologic cancers in patients with RA was increased compared to the general population (HR 3.58; 95% CI: 1.69, 7.60). Of solid malignancies, lung cancer was diagnosed in 29 patients in the RA cohort; the incidence was increased compared to the non-RA cohort with HR 1.97 (95% CI: 1.08, 3.59).

### 3.2. Risk Factors for Malignancy in RA

Characteristics associated with a high risk of any malignancy included smoking (HR: 1.60; CI: 1.09, 2.34) and erosions/destructive joint changes (HR: 1.47; 95% CI: 1.04, 2.09; Table 2). Smoking was associated with increased risk of not only lung cancer but also risk for any malignancy. The HR for any malignancy comparing current smokers to never or former smokers was 1.60 (95% CI: 1.09, 2.34); that of lung cancer was also increased 3.59 (95% CI: 1.23, 10.48). The risk of lung cancer in RA compared to non-RA was attenuated by adjustment for smoking status (HR: 1.48; 95% CI: 0.81, 2.70). The risks for any malignancy (HR: 1.26; 95% CI: 0.99, 1.62) and hematologic cancers (HR: 3.48, 95% CI: 1.63, 7.41) were also somewhat smaller after adjustment for smoking status.

There were no apparent calendar time trends in the occurrence of malignancies among the RA or non-RA cohorts.

The use of glucocorticoids in patients with RA was associated with increased malignancy risk (HR 1.57; 95% CI: 1.04, 2.39). This association between glucocorticoid use and development of any malignancy persisted after additional adjustment for ESR at RA incidence, rheumatoid factor positivity, and current smoking (HR: 3.59; 95% CI: 1.03, 2.40).

Methotrexate use was not associated with overall increased malignancy risk in patients with RA (HR: 1.04; 95% CI: 0.72, 1.50). Other DMARDs as a group were not associated with an increased risk for malignancy, although the risk of lung cancer was numerically but not significantly increased (HR: 2.38; 95%; CI: 0.91, 6.22). As well, malignancy risk was not associated with use of biologics (primarily antitumor necrosis factor agents), glucocorticoids or anti-inflammatory analgesics (Table 2). The risk of lung cancer was further attenuated by additional adjustment for erythrocyte sedimentation rate (ESR) at RA incidence, rheumatoid factor positivity, and current smoking (HR: 1.50; 95% CI: 0.58–3.92). The malignancy risk was not increased in this RA cohort with the use of biologics (HR 0.69; CI: 0.36, 1.35).

## 4. Discussion

This retrospective population-based cohort study examined the incidence of malignancy in a nonreferral community-based population with RA and the risk for individual malignancies, including specific solid tumors, lymphomas, leukemias, and skin cancers (NMSC and melanoma). Excluding NMSC in the risk estimation revealed an increased risk of cancer in patients with RA; however, when NMSC were included, the overall cancer risk was not increased.

In this study, the overall risk of malignancy in patients with RA was not associated with DMARDs or biologic response modifiers, principally TNF inhibitors. The use of glucocorticoids was associated with increased risk of any malignancy, but there was no apparent association between glucocorticoids and hematologic cancers or lung cancers. The association between glucocorticoid use and development of any malignancy persisted after additional adjustment for ESR at RA incidence, rheumatoid factor positivity, and current smoking. The reason for this association is not certain. However, given the observational nature of our study, glucocorticoid use may be confounded with more severe disease and increased inflammatory burden, which may be associated with malignancy risk.

The overall increased risk of cancer was largely driven by the increased risk of hematologic cancers. A link between lymphoma and RA was first reported from a medical record linkage study in 1978 [12]. Subsequently, a considerable body of evidence has emerged that supports RA and RA disease activity as pathogenic factors in the development of lymphoma [2, 3]. In a meta-analysis of 21 publications from 1990 to 2007 on the risk of malignancy in patients with RA, the risk of lymphoma was increased approximately twofold (SIR 2.08, 95% CI 1.8, 2.39), with a greater risk of both Hodgkin's and non-Hodgkin's lymphoma [13]. Lymphoproliferative malignancies are also increased in patients with extra-articular disease manifestations such as Felty's syndrome [14] and secondary Sjögren's syndrome [15].

Pooled data from 74 randomized controlled trials showed that TNF inhibitors were associated with an increase in risk of NMSC beyond the risk associated with RA alone [16]. Several large observational studies have supported this finding [17, 18], but others have not [19–21]. In a recent population-based cohort study conducted in Sweden, the risk of squamous cell cancer (SCC) and basal cell cancer (BCC) was evaluated in patients with RA naïve to biologic drugs, in patients starting TNF inhibitors treatment, and in the general population. This study demonstrated a 20% increased risk of BCC and a near doubled risk of SCC in patients with RA compared with the general population. For patients treated with TNF inhibitors compared with those naïve to biologics, BCC risk was moderately increased, but the increase was not significant after adjustments for demographic and comorbidity variables. Limitations of that study include that the authors were unable to adjust for severity of disease. People with more severe arthritis could be more likely to receive TNF inhibitor. If severity of arthritis is related to risk of NMSC, then this outcome is confounded by indication. Patients were not randomly assigned to treatments in this observational study, so any excess risk could be due to increased severity of disease rather than treatment [22].

One of the studies conducted among 13,001 patients using the US National Cancer Institute SEER (Surveillance, Epidemiology, and End Results) database revealed increased risk for skin cancers with biologic therapy, but not for solid tumors or lymphoproliferative malignancies. These associations were consistent across different biologic therapies [17]. Another study from The South Swedish Arthritis Treatment Group register (SSATG) evaluated the risk of malignancy in patients who had undergone TNF inhibitor therapy. This study demonstrated that patients receiving conventional RA treatment had an increased overall tumor risk compared with the background population. Possible additional increased risk for lymphoma associated with TNF inhibitors was also reported in few cases [23].

The risk for lung cancer was increased in the current study. An increased risk of lung cancer has been reported in individual studies [4], as well as in the meta-analyses [13]. This may be related to an increased risk of RA in smokers described in population-based prospective cohort studies [24, 25]. Conversely, in a study of patients with RA in the US veterans' population, the risk of lung cancer was increased by 43% compared with the general population, even after adjustment for tobacco and asbestos exposure [26].

Strengths of this study include its population-based design and complete medical record review. The Rochester Epidemiology project affords the ability to include both patients with RA and age- and sex-matched comparator subjects living within the same community, reducing biases of referral populations. The average follow-up of 14 years in this study is much longer than the majority of other retrospective studies [13]. The length of follow-up in the current study permits assessment of long-term risk and secular trends in cancer development compared to most studies which are of shorter duration.

Limitations may include the fact that the population of Olmsted County is predominately Caucasian; however results of REP studies are generally applicable to other population cohorts [26]. In addition, there is some concern with the size of our cohort, 813 RA patients, ~7800 patient-years of follow-up. While our study was adequately sized to detect increased risk for any malignancy, it was under-powered to detect increased risks for cancer subtypes.

## 5. Conclusion

There was a small to moderately increased risk of malignancies excluding NMSC in RA patients; the risk was highest for hematologic cancers. Risk for lung cancer was also increased. The overall risk of malignancy in patients with RA was not associated with DMARDs or biologic response modifiers, principally TNF inhibitors. Cancer surveillance is imperative in all patients with RA.

## Figures and Tables

**Table 1:** Cumulative incidence rates of malignancy in 813 patients with rheumatoid arthritis in 1980–2007 compared to 813 subjects without rheumatoid arthritis.

Malignancy site/type^*∗*^	Number of events after incidence/index in RA/non-RA	Cumulative incidence at 10 years for RA patients (± SE)	Cumulative incidence at 10 years for non-RA subjects (± SE)	Hazard ratio^*∗∗*^ (95% CI)
Any malignancy excluding NMSC	143/118	11.8 ± 1.2	9.3 ± 1.1	1.32 (1.03, 1.68)
Any malignancy including NMSC	194/179	15.6 ± 1.4	14.3 ± 1.4	1.13 (0.92, 1.38)
Any solid malignancy	116/113	10.3 ± 1.1	8.8 ± 1.1	1.11 (0.85, 1.44)
Hematologic	28/9	1.5 ± 0.4	0.6 ± 0.3	3.58 (1.69, 7.60)
Head/neck	11/9	0.9 ± 0.3	0.9 ± 0.3	1.41 (0.58, 3.42)
Colon/rectal	9/9	0.8 ± 0.3	0.8 ± 0.3	1.08 (0.43, 2.72)
Lung	29/17	2.1 ± 0.5	1.1 ± 0.4	1.97 (1.08, 3.59)
Breast (among females)	24/29	1.8 ± 0.6	3.5 ± 0.8	0.95 (0.55, 1.63)
Bladder	4/10	0.4 ± 0.2	0.6 ± 0.3	0.46 (0.14, 1.49)
Melanoma	11/13	1.2 ± 0.4	0.8 ± 0.3	0.90 (0.40, 2.00)

NMSC	86/109	6.3 ± 0.9	7.5 ± 1.0	0.83 (0.63, 1.10)
Basal cell carcinoma	57/75	3.3 ± 0.7	5.0 ± 0.7	0.81 (0.57, 1.14)
Squamous cell carcinoma	56/69	4.2 ± 0.7	4.3 ± 0.7	0.90 (0.63, 1.29)

NMSC = nonmelanoma skin cancer; RA = rheumatoid arthritis; SE = standard error; CI = confidence interval.

^*∗*^No malignancies were observed in these sites/types: multiple myeloma, myeloproliferative syndrome, and myelodysplastic syndrome. Comparisons were not performed for the following sites/types with fewer than 5 malignancies per cohort (number of events after incidence in RA/index in non-RA): gastric (1/3), pancreatic (4/2), other digestive (2/5), other thorax (1/0), bone (1/0), soft tissue (1/4), ductal carcinoma in situ (6/4), ovary (2/1), other gynecological (4/4), kidney (2/5), other genitourinary (2/2), ophthalmologic (1/0), central nervous system (2/3), and other (2/2).

^*∗∗*^Adjusted for age, sex, and calendar year of RA incidence/index date. Cumulative incidence is adjusted for the competing risk of death.

**Table 2:** Risk factors for malignancy in 813 patients with rheumatoid arthritis (RA) at RA incidence and during follow-up.

Characteristic	Value^*∗∗*^	Hazard ratio^*∗*^ for any malignancy (95% CI)	Hazard ratio^*∗*^ for hematologic malignancy (95% CI)	Hazard ratio^*∗*^ for lung cancer (95% CI)
*Baseline characteristics*				
Age, mean (± SD)	55.9 (± 15.7)	1.64^†^ (1.44, 1.86)	1.98^†^ (1.44, 2.71)	1.91^†^ (1.41, 2.59)
Female sex	556 (68.4%)	0.70 (0.50, 0.99)	0.89 (0.39, 2.01)	0.61 (0.28, 1.30)
Calendar year of RA incidence, mean (± SD)	1995.6 (± 7.9)	1.06^†^ (0.83, 1.35)	1.64^†^ (0.86, 3.12)	1.06^†^ (0.60, 1.88)
ESR at index, mean (± SD)	24.8 (± 20.5)	0.99^†^ (0.90, 1.09)	0.89^†^ (0.70, 1.13)	1.05^†^ (0.87, 1.27)
Highest ESR in 1st year, mean (± SD)	32.7 (± 25.7)	1.00^†^ (0.92, 1.08)	0.93^†^ (0.77, 1.13)	1.03^†^ (0.88, 1.21)
Rheumatoid factor positive	539 (66%)	0.91 (0.64, 1.30)	0.79 (0.35, 1.77)	3.59 (1.23, 10.48)
Current smoker	178 (22%)	1.60 (1.09, 2.34)	0.38 (0.09, 1.63)	22.40 (8.48, 59.21)
Former smoker	271 (33%)	0.91 (0.63, 1.31)	1.60 (0.72, 3.56)	0.42 (0.17, 1.06)
Body mass index, kg/m^2^	27.7 (± 5.9)	1.00 (0.97, 1.03)	0.95 (0.88, 1.04)	0.95 (0.87, 1.03)
Obesity (body mass index ≥ 30 kg/m^2^)	244 (30.0%)	0.75 (0.50, 1.13)	0.81 (0.32, 2.05)	0.77 (0.31, 1.92)
*Time-dependent characteristics *				
BMI ≥ 30 kg/m^2^, ever	391 (48%)	1.03 (0.73, 1.45)	0.89 (0.40, 1.98)	0.61 (0.27, 1.36)
BMI < 20 kg/m^2^, ever	196 (24%)	0.83 (0.51, 1.34)	0.66 (0.19, 2.28)	3.12 (1.29, 7.55)
Alcohol abuse	69 (8%)	1.82 (1.07, 3.10)	—	7.89 (3.41, 18.22)
Rheumatoid nodules	272 (33%)	1.20 (0.83, 1.73)	1.95 (0.87, 4.37)	2.49 (1.14, 5.40)
Erosions/destructive changes	459 (56%)	1.47 (1.04, 2.09)	1.88 (0.81, 4.34)	2.65 (1.14, 6.16)
Severe extra-articular manifestation of RA	94 (12%)	1.41 (0.84, 2.35)	0.79 (0.19, 3.38)	1.99 (0.75, 5.29)
Large joint swelling	648 (80%)	1.06 (0.70, 1.60)	0.85 (0.33, 2.17)	3.42 (0.81, 14.54)
Joint synovectomy	89 (11%)	1.53 (0.93, 2.52)	0.69 (0.16, 2.98)	1.06 (0.31, 3.61)
Joint arthroplasty	174 (21%)	0.96 (0.61, 1.49)	0.27 (0.06, 1.18)	1.21 (0.50, 2.96)
Ever 3 ESR ≥ 60 mm/1 hr	114 (14%)	1.29 (0.78, 2.14)	0.62 (0.15, 2.67)	0.55 (0.13, 2.33)
Methotrexate	495 (61%)	1.04 (0.72, 1.50)	0.74 (0.31, 1.73)	1.49 (0.66, 3.39)
Other DMARD	580 (71%)	1.29 (0.88, 1.90)	1.35 (0.55, 3.31)	2.38 (0.91, 6.22)
Biologics	175 (22%)	0.69 (0.36, 1.35)	1.16 (0.32, 4.14)	0.34 (0.04, 2.61)
Glucocorticoids	650 (80%)	1.57 (1.04, 2.39)	0.87 (0.34, 2.22)	1.39 (0.54, 3.62)
COX-2 inhibitor	393 (48%)	1.09 (0.74, 1.60)	1.52 (0.65, 3.55)	1.10 (0.47, 2.58)
ASA (≥1950 mg/day; ≥3 mo)	338 (42%)	0.76 (0.51, 1.13)	0.53 (0.21, 1.34)	0.63 (0.26, 1.52)
NSAIDs for RA	738 (91%)	0.67 (0.40, 1.10)	1.17 (0.27, 5.04)	0.48 (0.18, 1.29)

^*∗∗*^Values are *n* (%) unless otherwise specified.

^*∗*^Adjusted for age, sex, and calendar year of RA incidence.

^†^Hazard ratio reported per 10 units increase.

ASA = acetylated salicylate; BMI = body mass index; CI = confidence interval; ESR = erythrocyte sedimentation rate; COX2 = cyclooxygenase 2; DMARD = disease modifying antirheumatic drugs; NSAID = nonsteroidal anti-inflammatory drug; RA = rheumatoid arthritis.
